# Spontaneous and heritable change in iridescent properties of bacterium *Tenacibaculum discolor str. IMLK18*

**DOI:** 10.17912/micropub.biology.001792

**Published:** 2025-09-30

**Authors:** Hooi Lynn Kee

**Affiliations:** 1 Biology, Stetson University, DeLand, FL, USA

## Abstract

Iridescence is a structural color phenomenon where varying-colored hues arise from the interaction of light with physical surfaces, rather than from chemical pigments. Iridescence was thought to be unique to specific organisms such as butterflies, peacocks and birds, but it has been recently reported in bacterial species. Here we isolated a previously uncharacterized iridescent marine bacterial species from the ocean in Woods Hole, MA. Under epi-illumination the iridescence changes from vibrant red/orange/yellow to green. How living bacterial cells produces iridescence is an intriguing question, given that the architecture that gives rise to iridescence in terrestrial organisms are typically made up of non-living biological material in fish scales, bird feathers, or the arthropod exoskeleton. Notably, when the bacteria are grown in liquid culture, they do not display iridescence, suggesting that the cells’ ability to self-organize into a community on solid surface determines their iridescence. Recent studies have shown gliding motility and physical sub-structures and arrangements are associated with iridescent properties in bacteria. Iridescent bacteria have also been investigated for their potential use as iridescent “bioink”. In this study, we report a surprising phenomenon, where a natural, spontaneous change in iridescence occurred during biofilm growth, giving rise to different iridescent color hues than normal. This is the first study describing the natural formation of iridescence mutants, not only in bacteria, but in any living system.

**Figure 1. Structural coloration of Tenacibaculum discolor IMLK18 biofilm with development of iridescence change:**
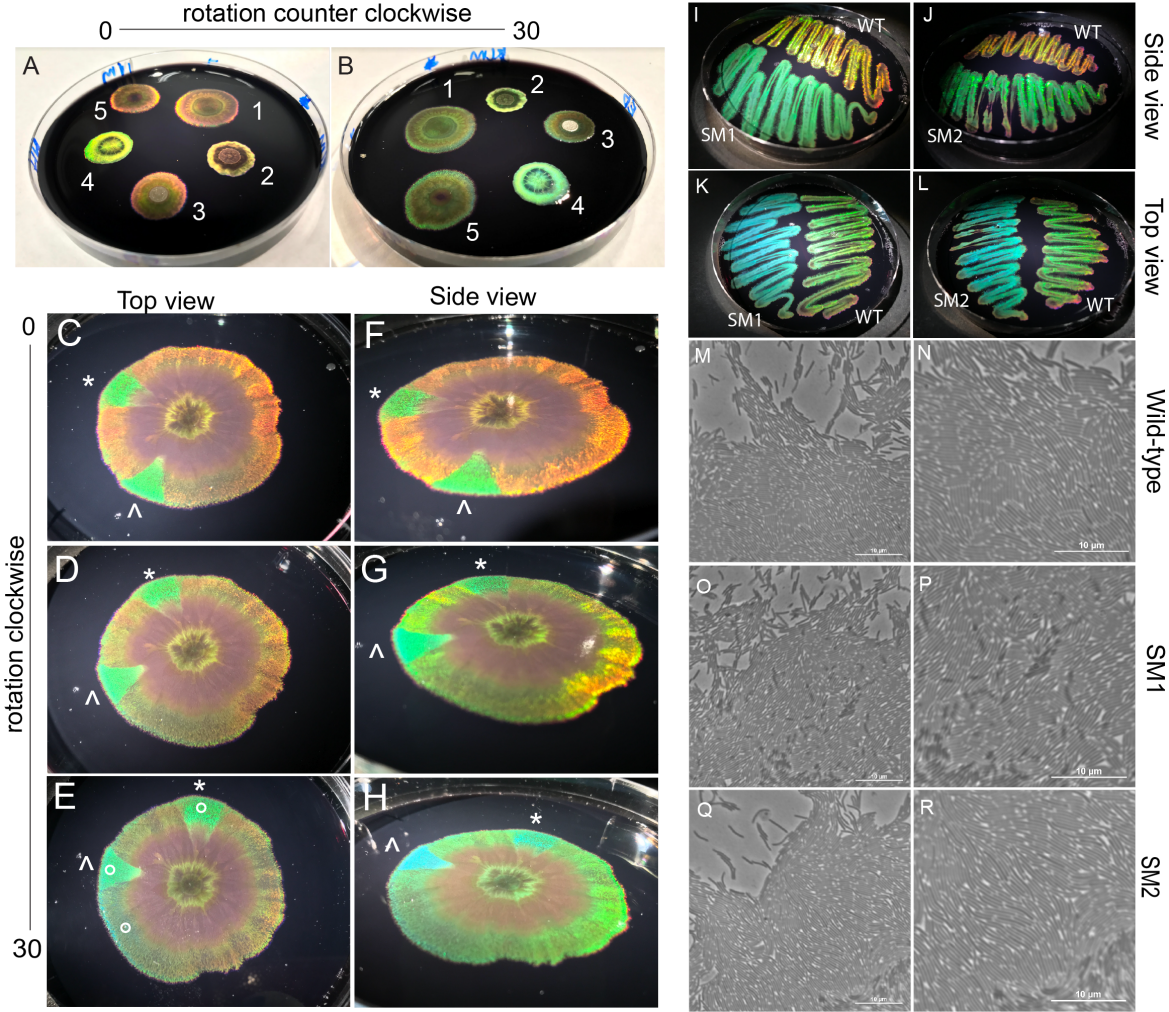
(A-B), Image of biofilms of different iridescent bacteria, numbered in white from 1 to 5, growing on a black plate. As the plate is rotated, iridescence colors change. (C-H) Images of wild-type biofilm 1 on 10cm plate, grown for 3 weeks, at room temperature on black agar plate. Triangular pie-shaped growth of altered iridescence is observed. C, D, E is photographed from above, while F, G, H are photographed from the side. The plate is rotated sideways to visualize the change in iridescence. * and ^ indicate the two different iridescent growths. The “o” indicates where cells were picked for genome sequencing. Bacteria of wild-type and spontaneous mutants 1 and 2 (SM1, SM2) were streaked out from original plate onto new plates. Differences in iridescence color are observed when looking at plates from the side (I, J) or from the top (K, L). (M-R) Bacteria were placed on an agar pad and imaged under 100x magnification (Nikon Ti-2E). The cells of wild-type (M,N), SM1 (O,P) and SM2 (Q,R) are rod-shaped and glide, and organize in clusters of aligned cells. (N, P, R) are more magnified images than (M,O, Q) where packing of individual cells can be seen. Scale bar, 10µm.

## Description

Color is a property ubiquitous in nature. Biological organisms have both pigmented color and structural color. With pigmented color, pigment molecules absorb light and reflect the color that is seen, whereas with structural color, highly ordered structural microarrangement of the physical surface scatters light in a way that allows us to visualize specific colors, as seen in butterflies and birds(Lloyd & Nadeau, 2021; Shawkey et al., 2009). For surfaces that display structural color, different hues can be visualized depending on the angle one looks at the surface, which is functionally the property of iridescence (Doucet & Meadows, 2009).

This report describes the isolation of an iridescent bacterial species, *Tenacibaculum discolor* , from the seawater off the beach near Trunk River in Woods Hole, MA (41° 32’ 4.853” N, 70° 38’ 27.745” W). Prior studies have described the characterization of iridescent strains collected from this site ( Figure 1A- B, bacteria labeled 2 and 5). Five different iridescent bacteria from this site are shown on a plate together in Figure 1. Thus, this site is a reliable habitat for isolation of iridescent strains and species belonging to this bacteria genus *Tenacibaculum* (Kee et al., 2019; Mickol et al., 2021). A dilution of the seawater sample was cultured on Sea Water Complete (SWC) agar at 25°C for one week. A single colony exhibited iridescence, and this was purified via three successive clonal picks. The iridescence exhibited was red-orange-yellow to green, as the angle of view changes ( Figure 1A- B, bacteria labeled 1). The 16srRNA gene sequence of this isolated strain was compared to the 16srRNA gene of other strains isolated and showed 100% sequence identity to the previously characterized strain, *T. discolor* IMLK18 (Kee et. al, 2019) and 96.91% similarity to *T. mesophilum strain ECR (* Mickol et al., 2021) *.*

Under phase microscopy, the isolated cells were all rod shaped and glide ( Figure 1M- Q). This is similar to previous studies of iridescent *Cellulophaga* bacteria, which have similar rod-shaped morphology and gliding behavior (Kientz et al., 2012). Furthermore, clusters of aligned cells were observed. In studies of *Cellulophaga lytica,* electron microscopy of fixed colony biofilms shows cells form self-organizing photonic crystals to create iridescence (Kientz et al., 2015, Sullivan et al., 2023). The bacteria from this study and previously studied iridescent bacteria species of genera *Tenacibaculum, Cellulophaga, Flavobacterium* and *Cytophaga* not only share the characteristic that surface translocation, specifically gliding motility, is associated with structural color, but collectively they also belong to the bacteria phylum Bacteroidota.

A biofilm of the iridescent strain was allowed to form from the center of a 10cm plate. As the bacteria grew, the emergence of two independent bacterial populations was observed ( Figure 1C- H) with distinct differences in iridescent properties. Specifically, the original isolated biofilm displayed iridescence in red and orange, while these new populations displayed bright green iridescence. Furthermore, clockwise rotation of the plate and the subsequent viewing angle change altered the iridescent color seen. The original isolated biofilm changed from red-orange to yellow to green, while the new populations displayed iridescence change from green to blue ( Figure 1F, G,H, extended data movie 1). To determine if this iridescent property was maintained over time, cells from regions within the new populations were picked, as well as cells from the original area of growth and streaked out on plates. Interestingly, such changes in iridescence appear to be heritable ( Figure 1I- L, extended data movie 2). Moving forward, these two populations will be denoted as spontaneous mutant 1 (SM1) and 2 (SM2).

To determine if SM1 and SM2 have acquired nucleotide changes in specific genes, a comparative genome analysis was conducted. From the original plate of biofilm growth, cells that displayed the original iridescence were picked, and cells with the new iridescent properties were also picked into liquid media to grow an overnight culture ( Figure 1E ). The wildtype genome consists of 74 contigs yielding a total length of 3,365,099 bp, an N _50_ contig size of 505175 bp. The average G+C content was 31.60%. The genome annotation was completed using Prokka (Seemann, 2014), which resulted in 3061 coding sequences (Lowe & Chan, 2016). To determine the nucleotide differences between SM1/2 and the wildtype bacteria, breseq analysis was used (Table 1) (Deatherage & Barrick, 2014). Notably, both iridescent mutants presented with the same T81A mutation in a hypothetical protein encoded by gene *POBFAJPO_02608* of unknown function. The hypothetical protein is 267 amino acids and appears to have a N terminal signal peptide sequence and non-cytoplasmic region predicted to be outside the membrane in the extracellular region (PFAM, InterPro). There are no predicted transmembrane domains. The protein has features of a Zinc-binding protease based on the Protein Homolog/analogy recognition Engine (Phyre) and InterPro analysis. A protein Basic Local Alignment Search Tool (BLAST) analysis shows similarities to proteins classified as a hypothetical protein in various species of *Tenacibaculum* genus, including 73.3% similarity to a putative zinc-binding metallopeptidase in *Tenacibaculum insulae* . A BLAST search in phylum Pseudomonadota shows 20-50% similarity to proteins classified as hypothetical proteins and putative zinc-binding metallopeptidases.

Additionally, each iridescent mutant had a second mutation in a different gene. SM1 had an A41V amino acid change in gene *uvrY_2* , which encodes response regulator protein UvrY. UvrY is the cognate response regulator for the BarA sensor kinase of *Escherichia coli (Alvarez et al., 2021; A.-K. Pernestig et al., 2000)* . In *E. coli* , UvrY and BarA form a two-component signal transduction system that functions to regulate carbon metabolism (A. K. Pernestig et al., 2003). In uropathogenic *E. coli* , deletion of *uvrY* leads to disruption of biofilm formation and swarming motility, possibly through regulating expression of genes associated with biofilm formation and virulence (Mitra et al., 2013).

SM2 had a Q226K amino acid change in gene *nreB_2* , which encodes for the oxygen sensor histidine kinase NreB. NreB has been previously characterized as a cytosolic oxygen sensing protein in staphylococci, working with NreC to regulate nitrate reductase and nitrite reductase operons (Kamps et al., 2004).

In a prior study on another reported iridescent strain, *Flavobacterium* strain Iridescent I (IRI), genes associated with altered optical properties were identified through transposon mutagenesis (Johansen et al., 2018). A few of their transposon insertions mapped to genes associated with gliding, thus concluding that gliding motility is necessary for cell organization and iridescence. Interestingly, other mutants from their study were mapped to non-motility genes, such as putative *trmD* *tRNA methyltranferase* and *spoT* related gene, suggesting that other genes may function to regulate iridescence. A recent genome wide association study of structural colored and non-structural colored bacterial isolates identified genes associated with structural color, which are involved in gliding motility, carbohydrate metabolism, and biosynthesis of compound uroporphyrin and cofactor pterins (Zomer et al., 2024).

Further work has investigated how external factors like algae polysaccharides influence structural color in bacterial colonies of IRI (Van De Kerkhof et al., 2022). Recent studies demonstrated that the intensity and colors of the iridescence of strain *Cellulophaga lytica* DSM 7489 can also be altered by external environmental changes such as media salinity and agar concentration (Sullivan et al., 2023). They showed that TEM analysis of cross sections of biofilms had differences in cell width measurements between red and green iridescent regions of a single biofilm. In our study, since the SM1 and SM2 mutants can be propagated on new plates with the same iridescence properties, it is unlikely that the change in iridescence properties in this study is due to external factors. Like prior studies, future experiments will use microscopy to investigate whether differences in cellular width and cell morphology also exist between the original and SM1/SM2 biofilms of this study.

In summary, this is the first report that describes the development of spontaneous and heritable changes to structural color properties without exogenous and genetic manipulation, not only in a living iridescent bacterium, but in any living system. Genomic analysis shows differences in nucleotides between original and iridescent mutant biofilms. The observation that two independent mutations at the same site in the same gene, the T81A mutation *POBFAJPO_02608* , in both SM1 and SM2 is intriguing. The change in iridescent phenotype may be due to these specific mutations identified in the study or could be due to other phenomenon that cause changes to behavior of bacterial populations, such as heritable epigenetic changes or loss of a plasmid. Currently, this *T. discolor* strain cannot be genetically manipulated, thus further studies will need to develop a system for genetics. Future experiments will work towards confirming whether the loci identified in this study play a role in structural color and iridescence of this bacterium. Potential experiments include determining whether expression of cloned version of wild-type genes in SM1 and SM2 reverses the iridescence change. The challenge will be to develop a system where exogenous genetic material can be introduced to this marine bacterium.

## Methods

Sea water was collected from the beach by Trunk River at Woods Hole. The sea water was diluted in a 1:10 dilution series and 100µl of each dilution was plated on seawater complete (SWC) plates. An iridescent colony was identified and streaked out three times on SWC plates to obtain a pure culture. The SWC agar plates contained: 1 liter Sea Water base, 5g tryptone, 1g yeast extract, 3ml 99% glycerol, 15g agar. SWC medium contained all the ingredients without the agar. Sea water base was made as follows: for 1 liter, 20gNaCl, 3g MgCl _2_ -H20, 0.15g CaCl _2_ -H20, and 0.5g KCl. Black plates were made by adding 1% Black Sheaffer Skrip Bottled Ink.

The formation of the biofilm of the iridescent strain was conducted on a 10cm black agar plate by placing 10µl of liquid culture in the center of the plate to allow radial growth over the course of 3 weeks. Images from Figure 1 (A-L) were captured on Apple iPhone 7 with camera settings of f/1.8 aperture, 1/20 shutter speed, and 3.99mm focal length. Plates were illuminated with a single gooseneck Microscope Illuminator Light (Nikon).

Genomic DNA was extracted from the pelleted cells of the liquid cultures using the Maxwell RSC PureFood GMP and Authentication Kit (Promega) and quantified using the dsDNA ONE Quantifluor kit (Promega) using the Quantas system (Promega). Libraries were generated using the Nextera DNA Flex Library Kit. Genome sequencing was conducted using Illumina (HiSeq 2500 2x 250 nt paired end reads). FastQC was utilized to assess quality of sequence reads. Genome assembly of 745950 paired reads was performed using SPAdes 3.13.1 (Bankevich et al., 2012).

This Whole Genome Shotgun project has been deposited at DDBJ/ENA/GenBank under the accession JBQWAV000000000. The version described in this paper is version JBQWAV010000000.

## Data Availability

Description: Structural coloration of Tenacibaculum discolor IMLK18 biofilm. Video of biofilm on 10cm black agar plate. Triangular pie-shaped growth of altered iridescence is observed.. Resource Type: Audiovisual. DOI: https://doi.org/10.22002/yatws-abs95 Description: Structural coloration of Tenacibaculum discolor IMLK18 SM1 biofilm. Video of biofilm on 10cm black agar plate. Differences between original and SM1 iridescence is observed.. Resource Type: Audiovisual. DOI: https://doi.org/10.22002/cjsnt-98j33
